# Sleep Enhances Recognition Memory for Conspecifics as Bound into Spatial Context

**DOI:** 10.3389/fnbeh.2017.00028

**Published:** 2017-02-21

**Authors:** Anuck Sawangjit, Eduard Kelemen, Jan Born, Marion Inostroza

**Affiliations:** ^1^Institute of Medical Psychology and Behavioral Neurobiology, University of TübingenTübingen, Germany; ^2^National Institute of Mental HealthKlecany, Czechia; ^3^German Center for Diabetes Research (DZD), Institute for Diabetes Research and Metabolic Diseases of the Helmholtz Center Munich at the University of Tübingen (IDM)Tübingen, Germany; ^4^Centre for Integrative Neuroscience, University of TübingenTübingen, Germany; ^5^Departamento de Psicología, Universidad de ChileSantiago, Chile

**Keywords:** sleep, memory consolidation, social recognition, spatial context, episodic memory

## Abstract

Social memory refers to the fundamental ability of social species to recognize their conspecifics in quite different contexts. Sleep has been shown to benefit consolidation, especially of hippocampus-dependent episodic memory whereas effects of sleep on social memory are less well studied. Here, we examined the effect of sleep on memory for conspecifics in rats. To discriminate interactions between the consolidation of social memory and of spatial context during sleep, adult Long Evans rats performed on a social discrimination task in a radial arm maze. The Learning phase comprised three 10-min sampling sessions in which the rats explored a juvenile rat presented at a different arm of the maze in each session. Then the rats were allowed to sleep (*n* = 18) or stayed awake (*n* = 18) for 120 min. During the following 10-min Test phase, the familiar juvenile rat (of the Learning phase) was presented along with a novel juvenile rat, each rat at an opposite arm of the maze. Significant social recognition memory, as indicated by preferential exploration of the novel over the familiar conspecific, occurred only after post-learning sleep, but not after wakefulness. Sleep, compared with wakefulness, significantly enhanced social recognition during the first minute of the Test phase. However, memory expression depended on the spatial configuration: Significant social recognition memory emerged only after sleep when the rat encountered the novel conspecific at a place different from that of the familiar juvenile in the last sampling session before sleep. Though unspecific retrieval-related effects cannot entirely be excluded, our findings suggest that sleep, rather than independently enhancing social and spatial aspects of memory, consolidates social memory by acting on an episodic representation that binds the memory of the conspecific together with the spatial context in which it was recently encountered.

## Introduction

Recognition of conspecifics is a fundamental cognitive ability in social species that combines innate and learning components. For example in humans, encoding and discrimination of faces relies on stimulus processing in predisposed cortical networks. However, new faces are learned and stored together with social and spatial features of the context in which they are encountered, and accordingly recognition of faces strongly depends on such context features (Dudas et al., 2005; Ison et al., 2015; Viskontas et al., 2016), and such binding of social experience in spatial context similarly occurs in rodents (e.g., Fellini and Morellini, 2013). In rodents, social recognition memory involves spontaneous exploratory behavior which makes the animal to explore a novel, unfamiliar conspecific longer than a familiar one. In fact, taking advantage of this innate behavioral preference for unfamiliar conspecifics, the relatively longer social investigation towards an unfamiliar vs. a familiar conspecific has been established as a standard measurement of memory for a previously encountered animal (Thor and Holloway, 1982; Engelmann et al., 1995; van der Kooij and Sandi, 2012; Lukas et al., 2013). The procedure requires a direct encounter between conspecifics which allows the animal to investigate both volatile and non-volatile fractions of an individual’s olfactory signature which is important because accessing only the volatile fraction is not sufficient to either encode or retrieve social recognition memory in rats (Noack et al., 2010; Engelmann et al., 2011).

Sleep has been shown to support consolidation of various types of memory in both humans and animals (Rasch and Born, 2013). However, little is known about the effect of sleep on the formation of social memory, which is typically assessed in recognition tasks. Two human studies reported an enhancing effect of post-learning sleep on the recognition of previously encountered face stimuli (Clemens et al., 2005; Wagner et al., 2007). Recognition performance was positively correlated with the amount of non-rapid eye movement (NonREM) sleep, consistent with the notion that NonREM sleep specifically benefits memories involving the hippocampal memory system (Diekelmann and Born, 2010). One study in rats revealed influences of the circadian rhythm on social learning. An increase in exploration time toward a novel juvenile conspecific was greater when the rats were tested during the rest phase than when tested during the active phase, and when the retention interval was extended from 30 min to 60 min (Moura et al., 2009), suggesting consolidation processes taking place during sleep might facilitate social recognition performance. However, to the best of our knowledge, the role of sleep in social recognition memory has not directly been examined in a rodent model, so far.

The present study aimed to test the effects of post-learning sleep on consolidation of social recognition memory in rats. We hypothesized that sleep enhances memory formation for conspecifics. A second goal was to explore whether the presumed consolidation of social memory interacts with the simultaneous consolidation of spatial memory. Indeed, in natural conditions social learning typically occurs embedded in a spatial context, and spatial memory is well-known to benefit from sleep (Inostroza et al., 2013; Oyanedel et al., 2014; Maingret et al., 2016). Moreover, memory formation in both domains shares common hippocampal circuitry, specifically in CA2 (Hitti and Siegelbaum, 2014; Schwarb et al., 2015; Alexander et al., 2016; Smith et al., 2016), rendering the possibility of interactions between the two domains occurring also during sleep-dependent consolidation. To simultaneously measure the effects of sleep on social memory and to dissociate contributions of spatial memory, we combined a standard social discrimination task with a modified radial arm maze in which the rat had direct access to both volatile and non-volatile fractions of each conspecific’s olfactory signature.

## Materials and Methods

### Animals

Thirty-nine 9–10 weeks old male Long-Evans rats (Janvier, Le Genest-Saint-Isle, France, 280–340 g) were used in this study. Eight 4–5 weeks old male juvenile rats of the same strain (Janvier, Le Genest-Saint-Isle, France, 85–125 g) served as social stimuli. Rats were housed in pairs and kept on a 12 h/12 h light/dark cycle (lights on at 6:00 h). They had free access to food and water throughout the experiments. All experimental procedures were performed in accordance with the European animal protection laws (Directive 86/609, 1986, European Community) and were approved by the Baden-Württemberg state authority (MPV 1/16).

### Experimental Task and Procedures

**Figure 1:**
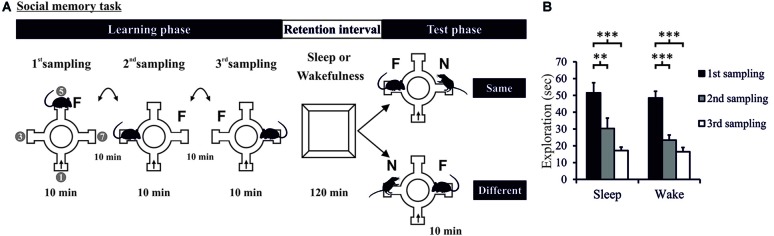
**(A)** Task and procedure. Social recognition memory was tested in a radial arm maze (four arms used). The Learning phase comprised three 10-min sampling sessions (separated by 10-min intervals) during which the experimental rat was allowed to explore a juvenile conspecific (i.e., familiar rat: F). The rat entered the maze always at the same arm (arrow). In each sampling session, the juvenile conspecific was placed at a different of the three remaining arms. The Learning phase was followed by a 120-min retention interval (spent in the retention box) in which the rat was allowed to sleep (Sleep group) or deprived of sleep (Wake group). Then, the 10-min Test phase took place during which the familiar rat (F) was presented along with a novel conspecific (N) at the opposite arm. For some rats, during the Test phase, the novel consepecific was located in the same arm as that of the familiar rat on the last sampling session (“Same”—upper panel). For other rats, the novel conspecific was placed in an arm different from that of the famliar rat during the last sampling session (“Different”—lower panel). “1”, “3”, “5”, “7” in left most maze denote the arms of the 8-arm radial maze used. **(B)** Exploration time during the Learning phase decreased across the three sampling sessions and did not differ between Sleep and Wake groups. ***p < 0.001, **p < 0.01.

The social recognition task was performed in a modified radial arm maze (Figure 1A) consisting of eight arms (14.5 cm wide, 35 cm long) placed radially around a central circular platform (11 cm wide, outer diameter 77 cm). Only four opened arms were used throughout the experiments (arm #1, #3, #5 and #7). The maze was made of wood and the outer part of the circular platform was surrounded by clear plexiglass acrylic sheets (18 cm height). At the end of each arm was a small platform (20 × 20 cm, not elevated) for placing the juvenile rat. The entire maze was lifted up 70 cm from the ground and surrounded by a white curtain. To allow navigation, distal cues were provided at the ceiling and attached at the arms of the maze, and the circular maze platform was surrounded by clear plexiglass such that the rat during navigation could see the whole maze including further proximal cues. During the sleep and wake retention intervals between the Learning and Test phase the animal was kept in another “retention box” (35 × 35 cm wide, 45 cm height) which was made of plastic and provided some bedding materials.

The experiments were performed during the light phase of the day (7:00–14:00 h). After 7 days of animal handling, habituation sessions were performed for five consecutive days (1 session per day). An opaque plastic basket with a lid was used to transport the adult rats to the experimental room and to the maze. The adult rats were allowed to explore freely in both the maze and retention box for 10 and 120 min, respectively. The experimenter left the room right after the rat was introduced to the maze, like in the experiment proper. To restrict movement of the juvenile rats to the square platform (further referred to as “juvenile zone”), they wore a harness made of latex, with a leash (30 cm) that was fastened to the end of the radial arm (Supplementary Figure 1). The juvenile was able to freely move within the juvenile zone but could not leave it. The juvenile rats were habituated to wearing the harness (also in the presence of an adult conspecific) and to stay in the juvenile zone over five consecutive days (10 min/day), whereby the arm used was changed across sessions. The habituation sessions for the adult and juvenile rats were performed separately.

One day after the habituation sessions, the Learning phase started with three 10-min sampling sessions. In each sampling session the adult rat was allowed to explore the juvenile rat which was presented at three different locations in the maze (Figure 1A). The starting point of the adult rat remained the same throughout the experiment. During the 10-min intervals between sampling sessions, the adult rats were kept in new cages with fresh bedding materials in a separate room. They were also habituated for this procedure during the habituation sessions. The Learning phase was followed by a 120-min retention period of Sleep (*n* = 18) or wakefulness (Wake, *n* = 18). During the sleep retention interval, the rat was left undisturbed in the retention box. The animal’s behavior was video-recorded, and sleep was assessed offline. In the wake retention interval the animal was deprived from sleep by gently handling (tapping on the retention box, or if necessary shaking the cage; Colavito et al., 2013). No intense stimulation was used to minimize stress. Arousal-interventions were introduced whenever the animal closed their eyes (with or without sleep posture) and was immobile for more than 5 s. In general, the rate of arousal-intervention to prevent the animal from sleeping during the 120-min sleep deprivation interval was <10 per hour, the overall number of arousals introduced per animal was <20. In fact, several previous studies showed that sleep deprivation with this procedure for a short period does not promote stress or anxiety, nor does it alter spontaneous motor activity or recognition memory, compared to undisturbed control animals (Kopp et al., 2006; Palchykova et al., 2006; Vecsey et al., 2009; Hagewoud et al., 2010a,b; Binder et al., 2012; Inostroza et al., 2013; Melo and Ehrlich, 2016).

During the 10-min Test phase following the retention period, a novel juvenile rat was presented along with the familiar rat, both at two opposing arms. The sequence of the juvenile rat’s locations in the Learning phase and the locations of the novel and familiar juveniles in the Test phase were randomized across rats but, kept balanced between Sleep and Wake conditions (Supplementary Tables 1, 2). The juvenile rats were littermates and they were housed in pairs. One hour before the Learning phase the juvenile rats were kept individually in new cages with fresh bedding materials outside the experimental room to avoid any contaminating odors. After the Learning and Test phase the maze and juveniles’ harness were cleaned with water containing 70% ethanol. The exploratory behavior of the adult rats was recorded by two video cameras placed on the two closed arms adjacent to the juvenile rat’s zone. Another camera was attached to a ceiling at the center of the maze to record the rats’ navigation through the maze. The recorded behavior was analyzed offline by an experienced researcher blind to the experimental condition using the ANY-Maze tracking software (Stoelting Europe, Dublin, Ireland).

### Data Analysis

*Sleep* during the retention interval was assessed using standard visual procedures (Kelemen et al., 2014). Sleep was scored whenever the rat showed a typical sleep posture and stayed immobile for at least 10 s. If brief movements interrupted sleep epochs by <5 s, continuous sleep was scored. The validity of this visual scoring procedure was demonstrated in previous studies in rats and mice, as well as in our own lab, consistently providing an agreement with conventional EEG/EMG based scoring of sleep of greater 92% (Van Twyver et al., 1973; Pack et al., 2007; Borquez et al., 2014). In animals of the Sleep group, the average sleep duration during the 120-min sleep retention interval was 49.22 ± 4.40 min (sleep-onset latency: 40.17 ± 3.86 min). Exploratory analyses did not reveal any significant correlation between sleep parameters (sleep latency, duration) and any of the performance scores during the Test phase.

In the *social recognition* task, exploratory activity was defined by the adult rat approaching the juvenile rat (to <2 cm) and sniffing the juvenile’s body surface irrespective of body area (Popik et al., 1991; Noack et al., 2010). During the Learning phase, the decrease in time spent exploring the (same) juvenile across the three sampling sessions served to confirm that the adult rat learned to discriminate the conspecific. Learning was defined by the decrease in exploration time from the first to the third session (Time exploring the juvenile rat during the 1st Sampling − Time exploring the juvenile rat during the 3rd Sampling) × 100/Time exploring the juvenile rat during the 1st Sampling]. Only animals reaching a learning criterion of >33% were included for further analyses. The learning criterion was introduced to reduce variance in the behavioral expression of memory.

In the Test phase, *social recognition memory* was measured by the animal’s preference to explore the novel rat, i.e., [(Time exploring the novel rat/Time exploring both rats) × 100]. Total exploration time spent with the juvenile rats was also analyzed. Additionally, preference to spend time in the novel rat’s zone was calculated by the formula: [(Time spent in the novel rat’s zone/Time spent in both rats’zone) × 100]. Statistical comparisons concentrated on: (i) cumulative scores for 10-s intervals across the initial 3-min interval of the Test phase and, to assess slower dynamics; on (ii) cumulative scores for 1-min intervals across the entire 10-min Test phase (Note, for calculating cumulative scores, rather than the preference scores *per se*, only the time the animal spent with the novel and familiar juveniles, respectively, was cumulated for a given time interval). Latency to explore the novel and familiar rat was analyzed with reference to the start of the Test session.

To assess *spatial memory*, the exploration preference for the novel rat during the Test phase was calculated separately for four spatial sub-conditions depending on the retention conditions (Sleep and Wake) and the location of the novel juvenile rat with reference to the location of the familiar juvenile on the last (i.e., 3rd) session of the Learning phase: (i) Sleep rats which were exposed to the novel juvenile at the same location as that where they encountered the familiar juvenile on the last sampling session (“Sleep-Same”); (ii) Wake rats which were exposed to the novel juvenile at the same location as that where they encountered the familiar juvenile on the last sampling session (“Wake-Same”); (iii) Sleep rats which were exposed to the novel juvenile at a location different from that where they encountered the familiar juvenile on the last sampling session (“Sleep-Different”); (iv) Wake rats which were exposed to the novel juvenile at a location different from that where they encountered the familiar juvenile in the last sampling session (“Wake-Different”). Please, note the terms “Same” and “Different” always refer to the novel juvenile’s location (during the Test phase) with reference to the familiar juvenile’s position on the last sampling session.

Statistical comparisons of exploration preferences for the novel rat relied on analyses of variance (ANOVA) with a group factor Sleep/Wake and a repeated measures factor Time interval. To dissociate effects on spatial and social memory in the spatial sub-conditions, ANOVA were run on the exploration preferences containing the group factors “Sleep/Wake” and “Spatial location”, the latter reflecting whether the juvenile rat was located at the same or a different location as the location of the familiar rat in the last sampling session. *Post hoc* *t*-tests were used to specify significant main and interaction effects. To test whether the exploration preference for the novel rat was above chance level (50%), one-sampled *t*-tests (two-tailed) were used. To reduce Type I error probability, the latter tests were only calculated after ANOVA indicated a significant Time main effect or Sleep/Wake × Time interaction, and significance is reported only when a *p*-value <0.05 was revealed for clusters of at least three neighboring time points. All statistical analyses were performed using SPSS 21. Results are reported as the mean ± SEM. Estimates of effect size, i.e., Cohen’s *d* and partial eta squared (*η^2^*), respectively) were also provided for the significant terms.

## Results

### Performance during the Learning Phase

Analysis of exploration times during the Learning phase showed that the rats in both Sleep and Wake groups learned to recognize the same juvenile rat across the three sampling sessions (*F*_(2,68)_ = 57.231 and 25.451, *p* < 0.001, partial *η^2^* = 0.386, for ANOVA Session main effect, Figure 1B and Supplementary Figure 2), with no difference between Sleep and Wake groups (*F*_(1,34)_ = 0.784, *p* = 0.382, for ANOVA Group main effect, and *F*_(2,68)_ = 0.462, *p* = 0.632, for ANOVA Group × Session interaction effect). Exploration time also significantly decreased from the first to the second and to the third sampling session (*p* < 0.006, for all pairwise comparisons). Two rats showed learning performance below the criterion (of a 33% decrease in exploration time from the 1st to the 3rd sampling session) and were therefore excluded from further analyses; decreases in these rats were 1.27% and 7.65%. Another rat was excluded due to technical problem during the Test phase. A total number of 36 rats (Sleep: *n* = 18, Wake: *n* = 18) was included in the analyses of the Test phase.

### Social Recognition Memory

**Figure 2:**
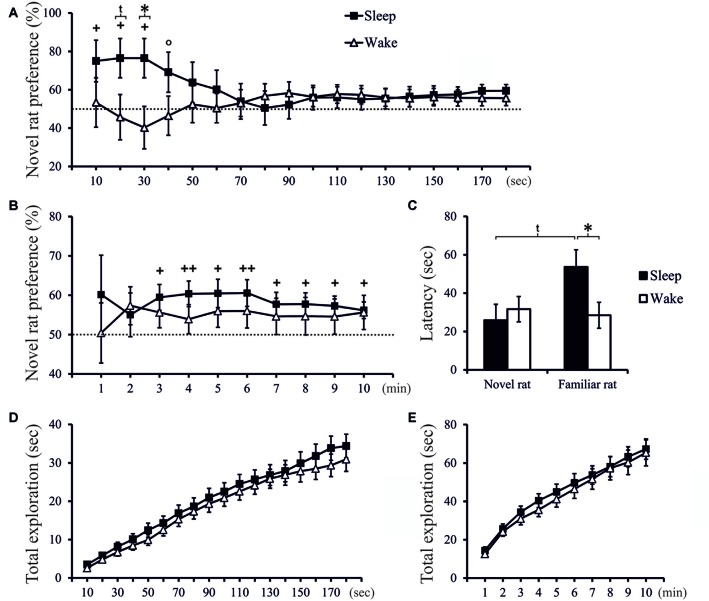
**(A)** Exploration preference for novel rat (in % of total exploration time) cumulated across 10-s intervals during the first 3 min of the Test phase, and **(B)** exploration preference cumulated across 1-min intervals across the total 10-min period of the Test phase, separately for the Sleep group (*n* = 18) and Wake group (*n* = 18). ^++^*p* < 0.01, ^+^*p* < 0.05, °*p* < 0.1, for tests against chance level (50%); *p < 0.05, ^t^*p* < 0.1, for difference between Sleep and Wake groups. **(C)** Latency (first approach) to explore the novel rat and the familiar rat. *p < 0.05, ^t^*p* < 0.1. **(D)** Total time spent exploring the juvenile rats (novel and familiar) cumulated across the first 3 min **(E)** and across the entire10-min Test phase. There was no significant difference in total exploration time between groups.

In the Test phase, only rats of the Sleep group, but not of the Wake group, displayed significant social recognition memory as shown by preferential exploration of the novel juvenile conspecific. Analysis of 10-s intervals revealed that sleep most profoundly affected exploration in the beginning of the Test phase. Exploration preference scores in the Sleep group were already above chance level within the first 30-s interval, and in this interval were also significantly different from exploration scores in the Wake group (*t*_(32)_ = 2.328, *p* = 0.026, *d* = 0.799; Figure 2A). To assess slower changes, we cumulated exploration preference scores across subsequent 1-min intervals. In the Sleep rats these scores reached significance from the 3rd min onwards (*p* < 0.05, one-sampled *t*-test), whereas in the Wake group scores remained at chance level (Figure 2B). Essentially the same results were obtained when exploration preference was scored based on the time the rat spent in each juvenile rat’s zone during the Test phase (see Supplementary Figure 3).

Latency to exploration of the familiar rat was significantly longer in the Sleep (53.67 ± 9.14 s) than Wake group (28.47 ± 6.92 s; *t*_(34)_ = 2.198, *p* = 0.035, *d* = 0.732), both groups did not differ in latency to explore the novel conspecific (Sleep = 25.90 ± 8.51 s, Wake = 31.66 ± 6.77 s; *t*_(34)_ = −0.529, *p* = 0.60, Figure 2C). Total exploration time spent with the juvenile rats (novel and familiar) was comparable between groups (*t*-test: *p* > 0.20 and 0.35 for the relevant 10-s and 1-min intervals, respectively, Figures 2D,E), indicating that the lack of novelty preference in the Wake rats was not a consequence of a non-specific reduction in exploration drive.

### Interaction between Sleep Effects on Social Recognition Memory and Spatial Memory

To discriminate interactions between the effects of sleep on social recognition and on spatial learning, exploration preferences in the Sleep and Wake groups were compared depending on whether the juvenile rat was located on the same spatial location (Sleep-Same, Wake-Same groups) or on a location different from that where the familiar rat was encountered in the last sampling session (Sleep-Different, Wake-Different groups). ANOVA on exploration scores cumulated across 10-s intervals confirmed the higher exploration preference scores in the Sleep than Wake group during the initial 30-s interval, as reported above (*p* = 0.026), and a main effect of Spatial location with a later onset, i.e., after 60 s, indicating preferential exploration toward the novel juvenile when it was placed on a location different from that of the familiar juvenile on the last sampling session (*F*_(1,32)_ = 4.265, *p* < 0.047, partial *η^2^* = 0.118, for Spatial location main effect between 60–90 s).

**Figure 3:**
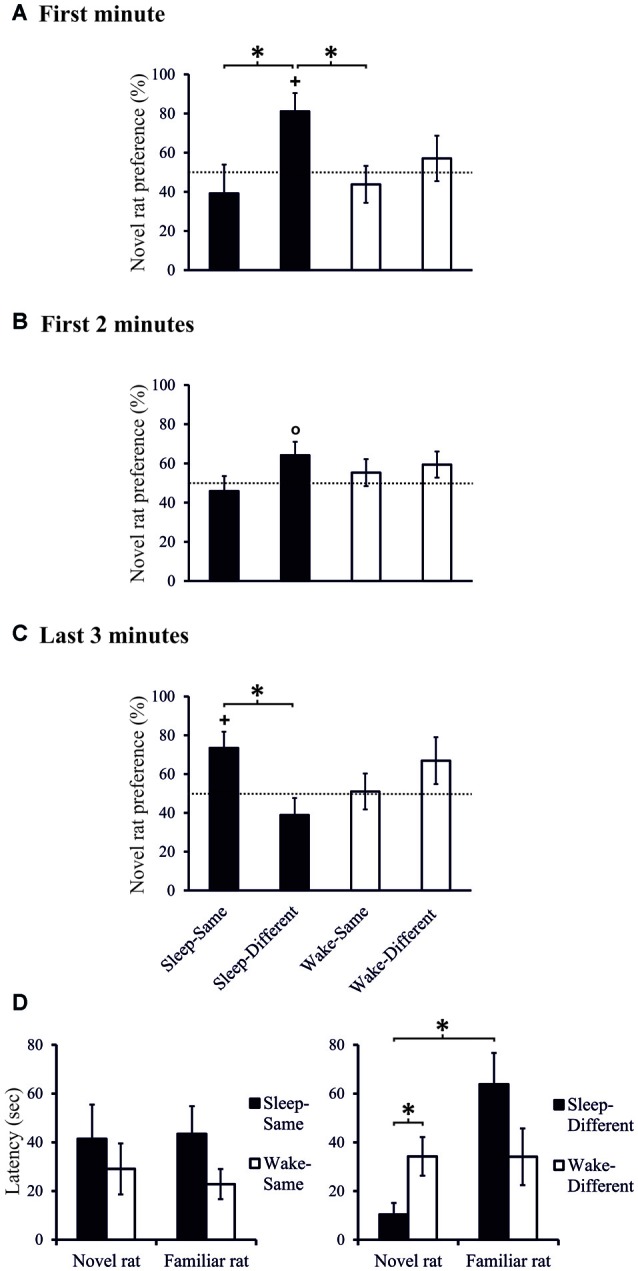
**Exploration preference for novel rat (in % of total exploration time) during (A)** the first minute, **(B)** the first 2-min interval, and **(C)** during the last 3-min interval of the 10-min Test phase, separately for the Sleep-Same, Sleep-Different, Wake-Same and Wake-Different groups. ^+^*p* < 0.05, °*p* < 0.1, for tests against chance level (50%); *p < 0.05, for difference between spatial sub-conditions. Note, only the Sleep-Different group shows above chance exploration preference in the first minute of the Test phase which also significantly differs from that of the Sleep-Same and Wake-Same groups (and from that of the Wake-Different group during the first 30-s interval, not shown). At the end of the Test phase, the exploration pattern in the Sleep groups reverses with higher exploration scores in the Sleep-Same than Sleep-Different group. *n* = 9 for all spatial sub-conditions during **(A)** the first minute and **(B)** the first 2-min interval of the Test phases, *n* = 8, 9, 9 and 8, for Sleep-Same, Sleep-Different, Wake-Same and Wake-Different groups, respectively during **(C)** the last 3-min interval of the Test phase. **(D)** Latency to explore (first approach) the novel and the familiar rat, separately for the Sleep-Same, Sleep-Different, Wake-Same and Wake-Different groups. *p < 0.05. Note, only the Sleep-Different group shows shorter latency to explore the novel rat in comparison to the familiar rat.

Separate analyses of the two spatial conditions revealed exploration scores that were above chance level, only in the Sleep-Different group, whereas in the other three conditions, i.e., the Sleep-Same, the Wake-Different and the Wake-Same rats, exploration scores failed to exceed chance level. In the Sleep-Different rats cumulated exploration scores reached significance during the first 70-s interval and again towards the end of the 3-min period of analysis. Considering that exploration scores remained at chance level in three of the four conditions, we used planned contrasts to directly test the hypothesis that sleep specifically enhances social recognition depending on where the novel juvenile was placed. Indeed, this analysis revealed that in the Sleep-Different group, exploration preference towards the novel juvenile across the first 60-s interval was significantly higher than in the Sleep-Same and Wake-Same groups (*t*_(16)_ = 2.270, *p* = 0.037, *d* = 1.070 and *t*_(16)_ = 2.659, *p* = 0.017, *d* = 1.253, Figures 3A,B) and, across the first 30-s interval, it was also significantly higher than that of the Wake-Different rats (*t*_(15)_ = 2.434, *p* = 0.028, *d* = 1.167). Moreover, in the Sleep-Different group latency to explore the novel juvenile the first time was significantly shorter than that for the familiar juvenile (10.38 ± 4.74 vs. 63.88 ± 12.82 s; *t*_(8)_ = 3.180, *p* = 0.013, *d* = 1.060) and was significantly shorter than the exploration latency for the novel rat in the Wake-Different group (34.23 ± 7.91 s; *t*_(16)_ = 2.438, *p* = 0.027, *d* = 1.149, Figure 3D). For all other groups exploration latencies were comparable for the familiar and novel juvenile (all *p* > 0.7). Overall, these data indicate that sleep robustly enhances exploration towards the novel conspecific depending on where this novel rat is encountered, i.e., only if this novel conspecific is encountered at a place different from that of the familiar rat in the last sampling session.

Notably, during the last 3 min of the 10-min test interval the sleep effect appeared to turn such that here exploration preference towards the novel juvenile was significantly greater in the Sleep-Same than in the Sleep-Different rats (*t*_(15)_ = 2.668, *p* = 0.018, *d* = 1.301, Figure 3C). Also, during these last 3 min, only the Sleep-Same rats showed above chance level exploration scores (*t*_(7)_ = 2.625, *p* = 0.034, *d* = 0.928, one-sampled *t*-test, *p* > 0.233 for the other 3 groups).

## Discussion

We examined the effect of post-learning sleep on consolidation of social recognition memory in rats. We found that sleep enhanced recognition of a conspecific as indicated by preferential exploration of the novel as compared to the familiar conspecific during the first min of the 10-min Test phase. We also found clues that enhanced consolidation of social recognition memory during sleep depends on spatial context features of the social episode encoded before sleep: only the rats of the Sleep group which encountered the novel conspecific at a place different from that of the familiar rat during the last sampling phase, i.e., the Sleep-Different group, but not the Sleep-Same group, expressed significant social memory during the first minute of the Test phase, and only this Sleep-Different group showed significantly enhanced social recognition memory during this 1-min period in comparison to all other groups, including the Sleep-Same group. This pattern of findings argues against a direct enhancing effect of sleep on social memory representations but is rather consistent with the notion that the consolidation effects of sleep on social and spatial aspects of a memory representation interact.

While there is evidence that sleep in rats can facilitate intermediate and long-term memory for social information like the transmission of food preference (Wooden et al., 2014), social recognition memory for conspecifics, has so far only been observed in adult rats with retention intervals of shorter duration, i.e., no longer than 30 min (Sekiguchi et al., 1991; Engelmann et al., 1995; Squires et al., 2006; Noack et al., 2010). Against this backdrop the present study provides first time evidence that the memory of conspecifics can last distinctly longer after sleep, i.e., a 2-h duration that pertains to so-called intermediate-term memory (Kesner and Hunsaker, 2010). The sleep effect expressed itself in the beginning of the Test phase within the first 30 s. In this interval, the Sleep group displayed exploration preference of the novel juvenile significantly above chance level, and the exploration preference for the novel conspecific was also significantly stronger if compared to those of the Wake group. Fittingly, in the Sleep rats latency to explore the novel conspecifics was shorter than to explore the familiar one, whereas such difference was not observed in the Wake rats. The latency of exploration might be related to the rat’s ability to detect the novelty of the conspecific based on its olfactory signature which seemed to be facilitated after sleep (Popik et al., 1991; Noack et al., 2010).

It might be argued that rather than effects of sleep, the observed differences in exploration preference between the Sleep and Wake animals during the Test phase reflect non-specific effects on memory retrieval, due to the fact that the Wake animals were tested immediately after the 120-min period of enforced wakefulness. However, in the Test phase, total exploration time spent with both novel and familiar juvenile rats, total time spent in the juvenile rat’s zone as well as the total number of entries into this zone (data not shown) were closely comparable between the Sleep and Wake animals. This makes it highly unlikely that the lack of social recognition memory in the Wake rats was a mere consequence of, e.g., a generally reduced motor activity or a decline in explorative drive, due to increased fatigue or a lack of attention in these animals after the extended wake period (Palchykova et al., 2009; Cho et al., 2010; Colavito et al., 2013). Likewise, stress as a consequence of depriving the rats from sleep, can be ruled out as a factor that substantially affected exploration behavior in the Wake group animals because we chose a rather short period of sleep deprivation (120 min) and sleep deprivation was established by the gentle handling procedure. These conditions are well-known to keep potential stress at a minimum and not to induce substantial increases in blood levels of the stress hormone corticosterone (Kopp et al., 2006; Palchykova et al., 2006; Vecsey et al., 2009; Hagewoud et al., 2010a,b; Melo and Ehrlich, 2016). Moreover, previous experiments of ours (Inostroza et al., 2013; Borquez et al., 2014) comparing effects of a slightly shorter 80-min retention interval of sleep deprivation with effects of a 80-min interval of spontaneous wakefulness (in the animals’ active period) did not reveal any difference in subsequent retrieval of hippocampus-dependent memories between these conditions, thus further excluding the possibility that impaired social recognition in our Wake animals would reflect adverse side effects of having the animals deprived from sleep before the Test phase. Also, both adult and juvenile rats were extensively habituated to the experimental setting which included habituation of the juvenile rats to being explored by an adult conspecifics. Thus, stress-related ultrasound vocalizations by the juvenile should not have substantially affected the adult rat’s exploratory behavior. Even if there was some vocalization by the juvenile rats, it should have equally affected performance of the Sleep and Wake group animals. Finally, both Sleep and Wake rats displayed comparable learning of the familiar conspecific as indicated by the decrease in exploration time spent with the juvenile rat across the three consecutive sampling sessions of the Learning phase. This assured that Sleep and Wake rats equally well learned to discriminate the familiar juvenile. Thus, the overall pattern of findings justifies to conclude that processes presumably taking place during sleep—rather than at learning or at retrieval—enhanced formation of social recognition memory in the Sleep rats. Nevertheless, this conclusion needs to be further scrutinized, e.g., by experiments directly controlling for possible differential effects on retrieval of sleep and wakefulness immediately preceding the retrieval test.

Our paradigm allowed us to test possible interactions between social recognition and spatial memory formation. During the three consecutive sampling sessions the adult rat was exposed to the juvenile rat presented at three different locations in the maze, and the decrease in exploration time across sampling sessions suggested that in both the Sleep and Wake group the rats were able to recognize the juvenile regardless of the location where it was located. Shifting the conspecific’s location across sessions is expected to foster the formation of a social representation that is quite independent of the places where the experimental rat encountered the juvenile during the Learning phase. Nevertheless, our results suggest that the formation of social recognition memory during sleep is modulated by spatial information, and depends on where the familiar juvenile was located on the last sampling session before sleep. After sleep, the adult rat showed a significant preference to explore the novel juvenile rat only when this novel juvenile was placed at a location different from that of the familiar juvenile during the last sampling session. Moreover, these Sleep-Different rats were not only the only group that displayed significantly shorter latencies to explore the novel than the familiar conspecifics, but their latency to explore the novel conspecific was also significantly shorter than that in the respective Wake-Different control group. Considering the distance between the novel and familiar rat’s zones, the latency of exploration is likely related to the rat’s ability to detect the novelty of the conspecific based on the non-volatile fraction of the juvenile rat’s olfactory signature. Overall, these effects of sleep revealed exclusively in the Sleep-Different group support the view that consolidation processes during sleep do not enhance the social representation of the conspecific *per se*, but that these processes act primarily on episodic-like representations (Kart-Teke et al., 2006; Inostroza et al., 2013) binding the social event into concurrent spatial contexts.

Interestingly, the Sleep-Same rats which encountered the novel juvenile at the same place as that of the familiar rat in the last sampling session, also formed significant recognition memory for the juvenile conspecific. However, this memory expressed only after a substantial delay in the last 3 min of the Test phase. This late onset of preferential exploration of the novel conspecific is difficult to explain, though it corroborates the view that social and spatial memory formation during sleep interact. It might be explained by a competing influence of spatial memory for the familiar conspecific’s location at the last sampling session which prevented an earlier expression of recognition memory for the juvenile. A previous study provided evidence for competition between two memory domains (item vs. space) in recognition memory (Haettig et al., 2011). In that study, mice failed to show preference for a novel object when the location of a familiar object was changed between learning and testing. Reversible inactivation of the dorsal hippocampus revealed that object recognition memory *per se* remained intact in this modified test context, suggesting that such competition affects primarily the expression of memory rather than the memory itself. Accordingly, it is possible that in the present study sleep independently enhanced spatial and social aspects of the sampling sessions experienced before, and that the different dynamics in the expression of exploratory preferences between the Sleep-Different and Sleep-Same group were merely due to the fact that in the latter group, placing the novel juvenile at the same location as that of the familiar juvenile during the last sampling epoch induced competition. However, this explanation is unlikely for the following reasons: rats of the Sleep-Different group expressed most pronounced exploratory preference in the very beginning, i.e., within the first minute of the Test phase and, thereafter quickly ceased to show preference behavior which is a typical dynamics for memory-driven exploratory behavior (e.g., Dellu et al., 1992; Dix and Aggleton, 1999; Chambon et al., 2011). By contrast, in the Sleep-Same group a significant exploratory preference for the novel juvenile emerged not until the seventh minute of the Test phase, i.e., long after any competing spatial exploration behavior should have ceased. Moreover, analysis of the Sleep-Same rats did not provide any evidence that these rats during the first minute of the Test phase, more strongly engaged in exploring the novel spatial aspects (i.e., the spatially displaced familiar juvenile).

We did not include a “non-social” control condition in our study (with object presented instead of juvenile conspecifics) which limits the interpretation of our findings as to whether they are specific to social experience. However, previous studies have revealed that the concurrent displacement of a familiar object can prevent expression of novel object preference (Haettig et al., 2011), very much agreeing with the present experiments using juvenile conspecifics instead of objects, and there is likewise evidence that sleep enhances the binding of an experienced object into its spatial context (Binder et al., 2012; Oyanedel et al., 2014). Also, performance during the Learning phase suggested that memory formation as tested in our task does not differently operate depending on whether social or non-social stimuli were employed. Rats of all groups learned to discriminate the (familiar) juvenile rat across the three sampling sessions as indicated by a most robust decrease in exploration time in the first minute of the 10-min sessions, similar to what is typically seen with non-social objects (e.g., Antunes and Biala, 2012). Interestingly, this decrease in exploration time during the Learning phase indicated that the rats learned to recognize the juvenile conspecific independent of its spatial location (which changed across sampling sessions) which is in stark contrast to the performance of the Sleep-Same animals which during the Test phase showed a distinctly delayed expression of social memory (i.e., not until the last 3 min of the Test phase). Thus, it appears that spatial context binding does not influence the formation of social recognition memory on the short-term (i.e., across the sampling sessions spaced 10 min apart) but rather emerges as an aspect of intermediate term consolidation during sleep.

It might also be argued that sleep promoted memory for a rule the rats learned across the three sampling sessions of the Learning phase, i.e., there is juvenile rat in the apparatus and it is always placed at a location different from that during the preceding trial. This view assumes that the rat learns to avoid arms of the maze that have been visited most recently. However, this explanation is very unlikely for two reasons: first, rats typically need much more trials than just the three sampling trials of the Learning phase in the present experiments to learn an alternation-like rule where rats requires to avoid the most recent response (e.g., Aggleton et al., 1986; Dudchenko, 2001). Second, if sleep had enhanced such learned rule—or if sleep had just enhanced spatial learning *per se*-, the Sleep-Same rats in the Test phase should have shown shorter latency to explore the displaced familiar juvenile and/or increased exploration time toward the displaced familiar conspecific in the beginning of the Test phase. However, exploration latencies and times in the Sleep-Same rats were very similar for the familiar and novel juveniles.

Our study aimed at establishing behavioral evidence that sleep affects the formation of social recognition memory and that this effect might interact with spatial episodic aspects of memory formation. Against this backdrop our study is limited as it does not provide any direct insight into the underlying neurophysiological mechanism of the effect of sleep. Recent studies have implicated a role of the hippocampal subfield CA2 in social recognition and contextual memory (Wintzer et al., 2014; Alexander et al., 2016), allowing for direct interactions between these aspects of memory to occur during sleep-dependent consolidation (Inostroza et al., 2013). Exposure to a conspecific induced remapping of CA2 place fields indicating how CA2 encodes social stimuli by modifying existing spatial representations. The CA2 region was found to be most sensitive in response to subtle changes of familiar spatial context, and remapping of CA2 ensembles for such spatial context also occurred when a familiar or novel conspecific was added to this context (Alexander et al., 2016). Together, this evidence suggests that during encoding the hippocampus integrates both social and spatial information into a single episode. Consequently, sleep might benefit social recognition memory by strengthening the integrated neuronal representation of the episode thereby enhancing social information as it is embedded in the spatial context most recently experienced. Our results support this view by demonstrating that after sleep the rats rapidly discriminated the familiar and novel conspecifics only in conditions most similar to the recently experienced episode (last sampling session), whereas displacing the familiar rat to a novel place attenuated social recognition. In this way the present findings might also be relevant for the understanding of social recognition in healthy humans as well as in patients with social-deficit disorders (like autism-spectrum disorders) that go along with specific alteration of sleep (e.g., Hirata et al., 2016; Mutluer et al., 2016).

## Funding

AS received a scholarship from the Development and Promotion of Science and Technology Talented Project (DPST), Thailand. EK was supported from the Ministerstvo Školství, Mládeže a Tělovýchovy (MŠMT) under the NPU I program (project Nr. LO1611). This research was supported by a grant from the Deutsche Forschungsgemeinschaft (DFG), SFB 654 “Plasticity and Sleep”.

## Author Contributions

EK, JB and MI designed the study. AS collected the data. AS and EK analyzed the data. AS, JB and MI wrote the article.

## Conflict of Interest Statement

The authors declare that the research was conducted in the absence of any commercial or financial relationships that could be construed as a potential conflict of interest.
