# Supplementary material for: Sleep Enhances Recognition Memory for Conspecifics as Bound into Spatial Context
Source: Front Behav Neurosci. 2017 Feb 21;11:28. doi: 10.3389/fnbeh.2017.00028 (PMC5319304; doi:10.3389/fnbeh.2017.00028)
Supplement: Supplementary file 1 [file SupplementaryMaterial.docx]

Supplementary Material

Sleep Enhances Recognition Memory for Conspecifics as Bound into Spatial Context

**Anuck Sawangjit, Eduard Kelemen, Jan Born, Marion Inostroza^*^**

*** Correspondence:** Marion Inostroza: marion.inostroza@uni-tuebingen.de

# Supplementary Data

Supplementary Material should be uploaded separately on submission. Please include any supplementary data, figures and/or tables.

Supplementary material is not typeset so please ensure that all information is clearly presented, the appropriate caption is included in the file and not in the manuscript, and that the style conforms to the rest of the article.

# Supplementary Figures and Tables

## Supplementary Figures


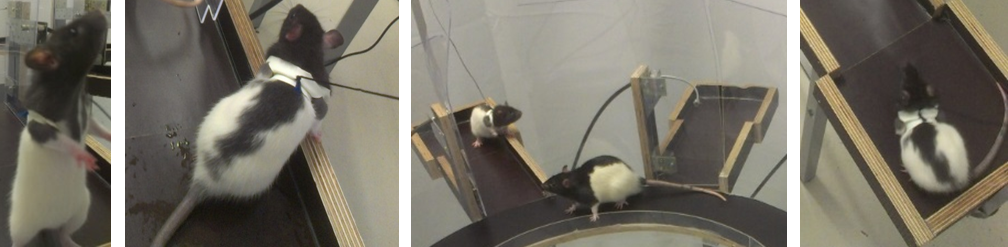


**Supplementary Figure 1.** Illustration of a juvenile rat wearing a harness in the juvenile’s zone. A harness was used to allow the adult rat to have direct interactions with the juvenile rat and to concurrently control the spatial context of the social stimulus. The juvenile rat was able to freely move within the juvenile zone but could not leave the zone. The procedure allowed the adult rat to perceive both volatile and non-volatile fractions of the individual’s olfactory signature.


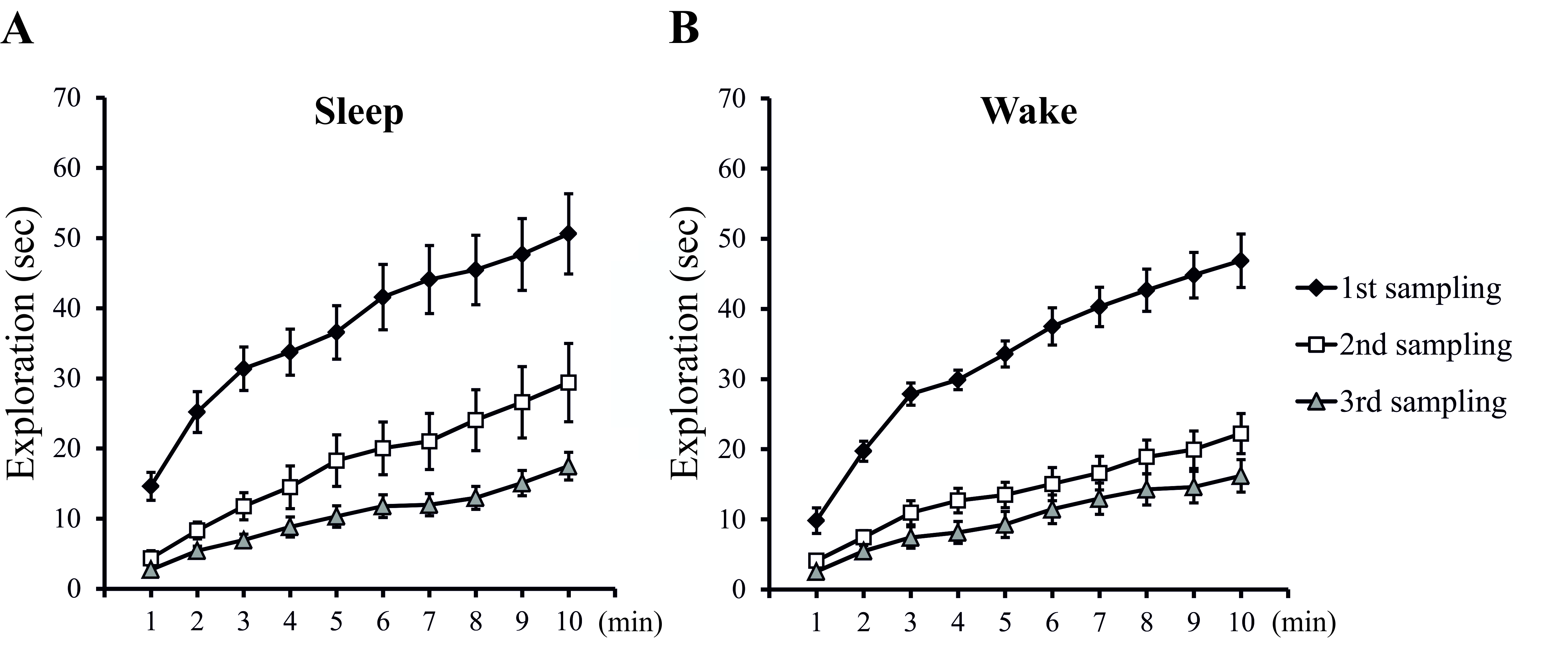


**Supplementary Figure 2.** Exploration time during the Learning phase cumulated across 1-min intervals, separately for (A) the Sleep group and (B) Wake group (n=18 for each sampling session in both groups). Note, significant decrease in exploration time from the first to the second and to the third sampling session was observed in both Sleep and Wake groups across 1-min intervals of the Sampling phase (p<0.006). There were no significant differences in exploration time between the second and the third sampling session, or between Sleep and Wake groups.

**
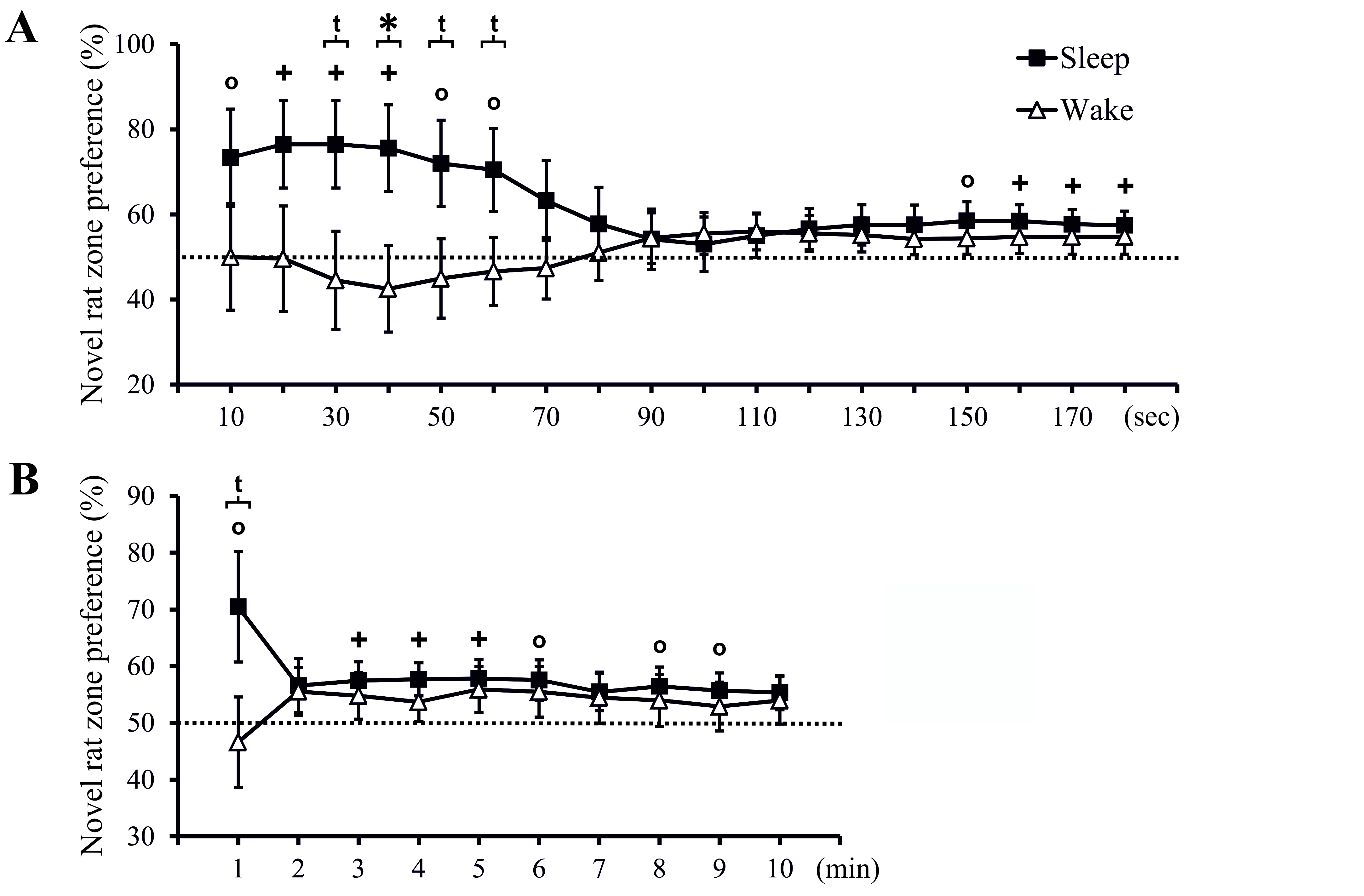
**

**Supplementary Figure 3.** Exploration preference assessed by the time spent in the novel rat’s zone (in % of total time spent in the zones of the novel and familiar rat) cumulated (A) across 10-sec intervals during the first 3 min of the Test phase, and (B) across 1-min intervals during the total 10-min Test phase, separately for the Sleep group (n = 18) and Wake group (n = 18). +: p<0.05, o: p<0.1, for tests against chance level (50 %); *: p<0.05, t: p<0.1, for difference between Sleep and Wake groups.

## Supplementary Tables

**Positions of the Juvenile Rat during the 3 Sampling Sessions of the Learning Phase**

| Position of juvenile rat - arm number - in session 1-2-3 | Number of juvenile rats | | | |
| --- | --- | --- | --- | --- |
|  | Sleep-Same | Sleep-Different | Wake-Same | Wake-Different |
| 3-5-7 | 3 | 3 | 3 | 3 |
| 7-5-3 | 4 | 1 | 3 | 1 |
| 5-3-7 | 1 | 2 | 2 | 1 |
| 5-7-3 | 1 | 3 | 1 | 4 |
| Total | 9 | 9 | 9 | 9 |

**Supplementary Table 1.** For placing the juvenile rat during the Learning phase 4 possible sequences of sampling locations were used, randomized across groups. One juvenile rat served as familiar or (in the Test phase) novel conspecific for 8-12 different adult rats, with a minimum of 24 hours between uses.

**Positions of Novel and Familiar Juvenile Rat during the Test Phase**

| Position of juvenile rat in the Test phase | Numbers of juvenile rats | | | |
| --- | --- | --- | --- | --- |
|  | Sleep-Same | Sleep-Different | Wake-Same | Wake-Different |
| 3 (Familiar) – 7 (Novel) | 4 | 4 | 6 | 5 |
| 7 (Familiar) – 3 (Novel) | 5 | 5 | 3 | 4 |
| Total | 9 | 9 | 9 | 9 |

**Supplementary Table 2.** During the Test Phase the novel and familiar juvenile rats were presented in two possible sequences of locations.
